# Prognostic and Predictive Implications of PTEN in Breast Cancer: Unfulfilled Promises but Intriguing Perspectives

**DOI:** 10.3390/cancers11091401

**Published:** 2019-09-19

**Authors:** Luisa Carbognin, Federica Miglietta, Ida Paris, Maria Vittoria Dieci

**Affiliations:** 1 Department of Medicine, University of Verona, 37126 Verona, Italy; luisa.carbognin@univr.it; 2 Division of Gynecologic Oncology, Department of Woman and Child Health and Public Health, Fondazione Policlinico Universitario A. Gemelli IRCCS, 00168 Roma, Italy; ida.paris@policlinicogemelli.it; 3 Department of Surgery, Oncology and Gastroenterology, University of Padova, 35128 Padova, Italy; federica.miglietta@iov.veneto.it; 4 Division of Medical Oncology 2, Istituto Oncologico Veneto IRCCS, 35128 Padova, Italy

**Keywords:** PTEN, breast cancer, prognosis, predictive role, treatment response, survival

## Abstract

The characterization of tumor biology and consequently the identification of prognostic and predictive biomarkers represent key issues for the translational research in breast cancer (BC). Phosphatase and tensin homolog deleted on chromosome ten (PTEN), the negative regulator of the proto-oncogenic phosphatidylinositol-3-kinase (PI3K)/protein kinase B (Akt) pathway, constitutes one of the most intriguing tumor suppressor genes involved in a series of biological processes, such as cell growth and survival, cellular migration and genomic stability. Loss of PTEN activity, due to protein, genetic or epigenetic alterations, was reported in up to almost half of BC cases. Recently, besides the role of PTEN in the pathogenesis of BC, investigated for over 20 years after the PTEN discovery, several retrospective and prospective translational studies, in the early and advanced setting, reported controversial results regarding the association between PTEN functional status and both clinical outcome and response to various BC treatments. This review explores the pre-clinical and clinical role of PTEN in BC with regard to the potential association of PTEN with prognosis and treatment response or resistance, underlying the complexity of the interpretation of available results and suggesting potential future perspectives.

## 1. Introduction

In the recent era of precision medicine, translational and clinical research have been matched together with enormous efforts in order to characterize tumors and customize treatment according to genetic, epigenetic or proteomic alterations. In the context of breast cancer (BC), the integration between technology, innovation and translational research represents a key issue to finally understand tumor biology and consequently to identify potential biomarkers of drug response or resistance. In this regard, phosphatase and tensin homolog deleted on chromosome ten (PTEN) represents one of the most frequently altered genes in human cancer, including BC. The role of PTEN as a key tumor suppressor gene, implicated in cell cycle progression, cell growth and survival in multiple tumor types, was discovered in 1997 by three independent research teams [1,2,3]. Specifically, PTEN encodes for dual-specificity lipid and protein phosphatase which downregulates the phosphatidylinositol-3-kinase (PI3K)/protein kinase B (Akt) signaling, a transduction pathway involved in a series of biological processes such as cellular motility, invasion, proliferation and survival. In particular, PTEN dephosphorylates the 3’-group of the phosphatidylinositol (3,4,5)-trisphosphate (PIP3), a pleiotropic second messenger that activates downstream signaling components, such as the protein kinase Akt, which stimulates downstream signaling pathways required for pro-survival processes. Thus, the dephosphorylation of PIP3 by PTEN turns off the PI3K/Akt pathway, inhibiting the growth and survival signals [4,5]. Moreover, a series of phosphatase-independent biological actions of PTEN in the cytoplasm and the nucleus have been described. These mechanisms include the binding of TP53, increasing TP53 stability and transcriptional activity, and the interaction with the APC/C (anaphase-promoting complex/cyclosome), enhancing the tumor-suppressive activity of the APC-CDH1 complex [6,7].

PTEN is also implicated in DNA repair and genome stability. Interestingly, it has been recently reported that in response to DNA damage, PTEN is phosphorylated on Tyr240, binds to chromatin and facilitates the recruitment of RAD51 to promote DNA repair, and thus, resistance to ionizing radiation therapy [8].

The loss of PTEN activity, identified in a series of primary and metastatic tumors such as breast cancer, leads into uncontrolled transduction of the PI3K signal. The PTEN inactivation is mainly due to somatic mutations including missense and nonsense mutations, monoallelic or biallelic deletion of PTEN gene locus, epigenetic silencing through promoter methylation, PTEN protein degradation and post-translational alterations of PTEN protein [9]. In particular, the post-translational modifications of wild-type PTEN (e.g., phosphorylation, mono- or poly-ubiquitination, sumoylation, oxidation and acetylation) are known to tightly regulate the function of the tumor suppressor as the result of altered subcellular localization, protein/protein interaction and/or phosphatase activity, independently of PTEN accumulation [10].

This process might explain some discrepancies related to PTEN expression levels, evaluated by next-generation sequencing or immunohistochemistry, and its biological functions.

Moreover, germline PTEN mutations are detected in inherited cancer syndromes, such as the Cowden syndrome characterized by the presence of multiple hamartomas and an increased risk for breast, thyroid, endometrium, kidney and colorectum malignancies [11].

Loss of heterozygosity at the PTEN locus was reported in nearly 40%–50% of breast tumors, whereas the loss of PTEN function due to PTEN mutations was detected in 5%–10% of BC cases, with frameshift representing the most frequent mechanism [12,13]. As for other solid tumors, epigenetic mechanisms of PTEN modulation have also been reported for BC [3,14]. Mutations in the PIK3CA gene represent another frequent mechanism of dysregulation of the PI3k/Akt pathway. Despite the fact that PTEN loss and PIK3CA mutations are commonly considered mutually exclusive events, a series of data reported concomitant alterations of the two genes in BC [15,16].

Although a large series of key data unravelling the role of PTEN in the pathogenesis of (solid) tumors have been currently reported to date, its contribution to BC progression in patients and its role as a potential predictor of resistance/efficacy to targeted agents present controversial issues and still require further investigation.

## 2. Preclinical Studies of Chromosome Ten’s (PTEN) Role in Breast Cancer (BC)

In the specific context of BC susceptibility, the role of PTEN was explored in PTEN-hypermorphic mouse models, expressing 80% of the normal levels of PTEN. In these mice, the slight decrease in PTEN expression seems to be sufficient to promote BC development and progression [17].

Several post-transcriptional mechanisms, including microRNA (miRNA) regulation, control PTEN expression. Recently, the RNA-binding motif 38 (RBM38), a RNA-binding protein and a target of the p53 family, was shown to regulate the PTEN expression in BC cells and tissues through its transcript stability. Thus, RBM38 may induce growth suppression by enhancing PTEN expression [18]. Moreover, a series of reports have pointed out the role of PTEN expression levels in stromal cells in a mouse model of BC. The PTEN genetic inactivation in stromal fibroblasts of mouse mammary glands may enhance the initiation and cancerous transformation of mammary epithelial tumors [19].

Besides the role of miRNAs in cell proliferation, a series of studies reported that certain specific miRNAs can be involved in drug-resistance [20]. In MCF-7 BC cells lines, the miRNA-222 may promote resistance to adriamycin through modulation of the PTEN/Akt/FOXO1 pathway [21].

With regard to targeted agents, pre-clinical data suggested a role of PTEN in treatment resistance to Trastuzumab. In HER2-positive (HER2+) BC cell-lines and xenografts, PTEN deficiency seems to contribute to Trastuzumab resistance by dephosphorylation and activation of SRC proto-oncogene nonreceptor tyrosine-protein kinase. Indeed, SRC seems to be hyperactivated in several Trastuzumab resistance models. Moreover, SRC activation and PTEN deficiency may be associated with poor outcomes in patients who received Trastuzumab-based therapy [14,22]. Thus, PTEN deficiency represents a potential predictor for Trastuzumab resistance.

Recently, a series of PI3K inhibitors has been investigated in BC pre-clinical and clinical settings. In pre-clinical studies, the predictive role of PTEN to PI3K inhibitors remains controversial. In this context, Tanaka et al. showed that PTEN deficiency was not associated with a response to CH5132799 (a class I PI3K inhibitor) in cell-lines and xenografts, including BC models. Conversely, PIK3CA mutation was suggested to be a predictor of a response [23]. A similar predictive role for PIK3CA mutation and not for PTEN loss of function was found in an in vitro study testing NVP-BEZ235 (a dual PI3K/mTOR inhibitor) [24]. In contrast, the analysis conducted by Stemke-Hale et al., suggested that PTEN loss in 12 hormone receptor-positive (HR+) BC cell lines was more predictive of response to LY294002—a potent PI3K inhibitor—than PIK3CA mutations [15]. Finally, Juric et al., reported that PTEN down-regulation was associated with resistance to the PI3K p110-alpha selective inhibitor BYL719, but not to the pan-PI3K inhibitor BKM120 in BC cell lines [25].

Interestingly, it has been suggested that PTEN loss may affect immune checkpoint regulation. Mittendorf et al., reported that PTEN knockdown increased cell-surface programmed cell death ligand 1 (PD-L1) expression and PD-L1 transcript in triple-negative BC cell lines, ultimately resulting, at the functional level, in decreased T-cell proliferation and increased T-cell apoptosis, as suggested by co-culture experiments [26].

## 3. Clinical Studies of PTEN’s Role in BC

Despite its potential biological relevance, the clinical utility of this biomarker remains controversial. Here, we reviewed available evidence on the prognostic and/or predictive role of PTEN status in BC patients, derived from biomarker analyses of patients enrolled in clinical trials.

### 3.1. Association with Prognosis

Although most studies failed to report an association between PTEN loss and prognosis in BC patients enrolled in clinical trials, emerging evidence suggests that lack or decrease in PTEN expression may be associated with worse outcomes in HR+/HER2 negative (HER2-) or HER2+ BC.

In detail, results from a biomarker analysis of 764 samples from the Breast International Group 1–98 (which randomized post-menopausal HR+ early BC patients to receive in the adjuvant setting tamoxifen, letrozole or the sequential treatment with letrozole followed by tamoxifen) revealed a non-significant trend towards increased risk of distant recurrence for patients with tumors harboring a PTEN mutation by next-generation sequencing, as compared with patients with wild-type PTEN (HR 1.67, 95% CI 0.69–4.06, *p* = 0.26) [27].

With regard to HER2+ BC, several randomized phase III trials did not report any significant association between survival and PTEN status assessed at protein level expression, either in the early [28,29] or advanced setting [30,31,32].

On the other hand, several authors have recently suggested that PTEN status may actually retain a prognostic role in this BC subtype, as highlighted by translational analyses from various prospective BC cohorts. Notably, Stern et al., reported that HER2+ BC patients treated with chemotherapy with or without Trastuzumab in the context of the adjuvant phase III BCIRG 006 trial who lacked PTEN expression exhibited poorer disease-free survival (DFS) and overall survival (OS), as compared to those with detectable PTEN staining by immunohistochemistry [33].

DNA analysis from the phase III APHINITY trial, randomizing patients with HER2+ early BC to receive adjuvant chemotherapy plus Trastuzumab and Pertuzumab versus chemotherapy plus Trastuzumab and placebo, revealed that PI3K/Akt/PTEN alterations (comprising PTEN homozygous deletion or any PTEN alteration) predicted worse outcome in terms of invasive DFS in a pooled analysis of both treatment arms [34].

### 3.2. Association with Treatment Response

Besides its potential prognostic role, PTEN status has also been suggested as a predictor of the response to different antitumor agents as revealed by translational analyses of several phase II and phase III clinical trials in HER2- (Table 1) and HER2+ BC (Table 2).

#### 3.2.1. mTOR Inhibitors

On the basis of the preclinical observation that PTEN loss may increase tumor sensitivity to mTOR inhibition [14,54], the predictive potential of PTEN in BC patients has been explored in the context of several clinical trials testing the mTOR inhibitor Everolimus for metastatic BC, with conflicting results. In detail, in the TAMRAD non-comparative phase II trial, HR+ metastatic BC patients were randomized to receive either Tamoxifen plus Everolimus or Tamoxifen alone. Biomarker analysis failed to report any predictive association between PTEN loss (assessed both by immunohistochemistry and next-generation sequencing analysis) and clinical benefit from the addition of Everolimus to Tamoxifen [35]. Similarly, in the BOLERO-2 phase III trial, randomizing HR+/HER2- metastatic BC patients to receive Everolimus plus Exemestane versus placebo plus Exemestane, PTEN mutations or low PTEN protein expression assessed on tumor samples were not associated with Everolimus benefit [36]. Conversely, the pooled analysis of the BOLERO-1 and BOLERO-3 phase III trials (testing the addition of Everolimus to Trastuzumab plus Paclitaxel and Trastuzumab plus Vinorelbine, respectively) revealed a potential predictive role of PTEN status with regard to Everolimus benefit. In particular, Andrè et al., reported that patients harboring low/absent PTEN tumor expression by immunohistochemistry or a hyperactive PI3K pathway (defined as either low/absent PTEN expression or PTEN mutations or PIK3CA activating mutations or AKT1 E17K mutations) experienced a significant progression-free survival (PFS) improvement with the addition of Everolimus to chemotherapy plus Trastuzumab, as compared to patients without these alterations [55].

#### 3.2.2. AKT Inhibitors

Interestingly, phase II trials evaluating the addition of AKT inhibitors to first-line Paclitaxel for triple-negative BC consistently reported an association between PI3K pathway deregulation (including PTEN alterations) and response to AKT inhibitors treatment both in advanced and early setting.

In particular, in the LOTUS trial, the addition of Ipatasertib to Paclitaxel significantly prolonged PFS of metastatic triple-negative BC patients (HR 0–60, 95% CI 0.37–0.98, *p* = 0.037). Patients were classified according to: PTEN expression by immunohistochemistry (PTEN-low or PTEN-high) and PIK3CA/AKT1/PTEN genomic alterations (based on the presence of either PIK3CA*,* AKT1 or PTEN mutations). In the subgroup of PTEN-low patients, there was no statistically significant difference in PFS between the two arms. However, in the PIK3CA/Akt1/PTEN-altered subgroup, the benefit of adding Ipatasertib to Paclitaxel was significant (HR 0–44, 95% CI 0.20–0.99). These data may suggest that the definition of PTEN functional status through mutations and copy number losses by next-generation sequencing may provide more biologically and clinically relevant information than the evaluation of PTEN protein expression by immunohistochemistry and that a comprehensive assessment of the PI3K pathway’s functional status may be of relevance rather than focusing on single alterations [37]. Similarly, in the PAKT trial, Schmid et al. reported that the PFS prolongation observed with Capivasertib plus Paclitaxel versus Paclitaxel plus placebo in triple-negative metastatic BC was mainly driven by the PIK3CA/Akt1/PTEN-altered population, since no beneficial effect from the addition of Capivasertib to Paclitaxel was observed in patients with PIK3CA/Akt1/PTEN non-altered tumors [39].

A similar predictive role of PTEN status has been suggested also in triple-negative early BC in the context of the FARLAINE trial, where the combination of Ipatasertib plus Paclitaxel has been compared to Paclitaxel plus placebo in the neoadjuvant setting. Oliveira et al., reported higher pathological complete response (pCR) rates with Ipatasertib both in PTEN-low (assessed by immunohistochemistry) and PIK3CA/Akt1/PTEN-altered (by next-generation sequencing) subgroups, as compared to patients without these alterations. In addition, higher complete response rates by magnetic resonance imaging were observed in the same biomarker-selected subgroups of patients [38].

The predictive role of PTEN status with respect to response to AKT inhibition has also been explored in HR+ metastatic BC. In particular, recently presented results from the phase Ib-II Faktion trial suggested that the activation of the PI3K/Akt/PTEN pathway (defined as activating PIK3CA mutations in tumor tissue/blood or PTEN null by immunohistochemistry in primary or metastatic tumor tissue) did not affect sensitivity to Capivasertib, since a beneficial effect from the addition of the Akt inhibitor to Fulvestrant in HR+ metastatic BC was observed in both PI3K/Akt/PTEN activated and non-activated tumors [40].

#### 3.2.3. Anti-HER2-Based Treatments

In HER2+ BC, low or absent PTEN expression has been reported in 20–25% of cases [56]. Although HER2 status remains the only recognized and validated predictive biomarker to select patients for anti-HER2-based treatment, a growing body of evidence suggests that biomarkers involved in the PI3K pathway may have a role in affecting the response to HER2-targeted therapies.

### 3.3. Single Anti-HER2 Blockade

#### 3.3.1. Trastuzumab

##### Early BC: Neoadjuvant Setting

Based on preclinical and clinical data suggesting that PTEN functional status may contribute to resistance to Trastuzumab-based therapy [10,57,58], such a potential predictive role of PTEN has been explored in the context of several clinical trials in HER2+ early BC, with non-univocal results.

In detail, Dave et al. reported significantly lower pCR rates after neoadjuvant Trastuzumab plus chemotherapy in HER2+ BC patients with low PTEN expression by immunohistochemistry, as compared to those with higher PTEN protein levels [43].

Consistently, it has recently been reported that in the HER2+ population of the GEPARQuattro phase III trial, low PTEN expression assessed by immunofluorescence was significantly and independently associated with lower pCR rates after neoadjuvant therapy with chemotherapy plus Trastuzumab in the overall HER2+ population and HR+/HER2+ subgroup [44].

##### Early BC: Adjuvant Setting

Two randomized phase III trials of chemotherapy +/− Trastuzumab for early BC failed to report an association between PTEN status and adjuvant Trastuzumab benefit in terms of either DFS [28,33] or OS [33].

#### 3.3.2. Lapatinib

##### Metastatic BC

The potential role of PTEN status in predicting response or resistance to Lapatinib-based therapy has been investigated in advanced BC in the context of several prospective cohorts of HER2+ BC patients, with conflicting results.

In a prospective global Capecitabine plus Lapatinib Expanded Access Program for patients progressing on Trastuzumab plus chemotherapy-based regimens, loss of PTEN by immunohistochemistry was associated with lower clinical benefit rates to Capecitabine plus Lapatinib than those with PTEN-positive tumors [59]. However, this observation was not confirmed in randomized phase II or III trials, where PTEN deficiency assessed by immunohistochemistry was not found to preclude a response to Lapatinib plus endocrine therapy or Lapatinib plus Paclitaxel in metastatic BC [31,45].

### 3.4. Dual Anti-HER2 Blockade

#### 3.4.1. Trastuzumab and Pertuzumab

##### Early BC: Neoadjuvant Setting

The potential role of PTEN status in predicting the benefit from HER2-dual blockade with Trastuzumab and Pertuzumab has been investigated in the context of neoadjuvant clinical trials. However, they did not report any correlation between PTEN protein expression (assessed by immunohistochemistry) and pCR rates after Pertuzumab plus Trastuzumab-based therapy [46,47].

##### Early BC: Adjuvant Setting

The recently presented translational analysis of the Aphinity trial (adjuvant treatment with either chemotherapy plus Trastuzumab and Pertuzumab or chemotherapy plus Trastuzumab and placebo) revealed a trend towards decreased benefit from the addition of Pertuzumab to adjuvant Trastuzumab plus chemotherapy in patients whose tumors harbored PI3K/Akt/PTEN alterations, such as PTEN homozygous deletion or any PTEN alteration by DNA analysis, thus further highlighting that a more comprehensive evaluation of PTEN status by genomic analysis may help with better capturing the biological relevance of PTEN in affecting response to anti-HER2-based therapy than the mere protein expression by immunohistochemistry [34].

##### Metastatic BC

The translational analysis of the Cleopatra study revealed that the survival benefit (PFS and OS) observed with the addition of Pertuzumab to Trastuzumab plus chemotherapy as first-line treatment for metastatic BC patients was independent from PTEN status [30].

#### 3.4.2. Trastuzumab and Lapatinib

##### Early BC: Neoadjuvant Setting

Although a positive association between low PTEN expression and pCR rates after neoadjuvant therapy with Lapatinib plus Trastuzumab and chemotherapy was initially suggested by Dave et al. [39], available data from phase II and III neoadjuvant clinical trials testing chemotherapy plus single or dual anti-HER2 blockade with Trastuzumab and Lapatinib failed to demonstrate significant differences in pCR rates according to PTEN status overall and per treatment arm [29,48,49,50].

In the context of the TBCRC 006 trial, low PTEN expression has been counterintuitively associated with lower pCR rates after dual HER2 blockade with Lapatinib and Trastuzumab without chemotherapy, as compared to higher PTEN expression [51].

Although these data highlight that solid evidence is missing on the actual role of PTEN in affecting the response to HER2 dual blockade, available evidence suggest that the PI3K/Akt pathway may actually retain a role in affecting the response to anti-HER2-based therapy. Indeed, a recent pooled analysis of almost 1000 HER2+ early BC patients from five randomized clinical trials of neoadjuvant treatment with chemotherapy in addition to either Trastuzumab, Lapatinib or the combination of them, reported lower pCR rates in PIK3CA mutant group as compared to the wild-type group, especially in the HR+ subpopulation and in patients receiving the dual HER2 blockade [60]. These data emphasize that a further deepening of the potential predictive value of the PI3K/Akt pathway with regard to anti-HER2-based therapy might be of great interest.

#### 3.4.3. Trastuzumab-emtansine (TDM1)

Biomarker analyses of randomized trials evaluating TDM1 for the treatment of HER2+ metastatic BC consistently report that PTEN was not predictive for TDM1 benefit [32,53]. However, a trend towards a more pronounced PFS benefit in patients with decreased or absent PTEN protein expression and treated with TDM1 relative to Capecitabine-Lapatinib has been reported, as compared to patients with higher PTEN expression levels [52].

Taken together, the available data, although preliminary, seem to generate the hypothesis that PTEN loss may potentially preclude response to Trastuzumab- or Lapatinib-based therapy, but not to TDM1.

#### 3.4.4. Immunotherapy

As suggested by preclinical models, PTEN loss may induce PD-L1 overexpression, thus hinting at a potential role of PTEN in affecting the response to immune-checkpoint inhibition.

Interestingly, Sousa et al. investigated PTEN genomic status in triple-negative metastatic BC patients treated with PD1/PDL1 immune checkpoint inhibitors—mostly associated with chemotherapy—in the context of several clinical trials (NCT02447003, NCT01375842, NCT02513472, NCT01633970) at Dana Farber Institute, reporting that PTEN alterations (mutations and 1 or 2 copy number loss) were significantly and independently associated with lower overall-response rate and worse PFS and OS rates with respect to patients without these molecular alterations [61].

#### 3.4.5. Endocrine Therapy

In a randomized non comparative phase II trial of Anastrozole or Fulvestrant as neoadjuvant endocrine therapy for post-menopausal HR+ BC patients, responders were found to harbor a significant overexpression of PTEN-related encoding gene (TPTE), as compared to non-responders, and this upregulation was maintained even after months of neoadjuvant endocrine therapy. Of course, this observation warrants further investigation [42].

#### 3.4.6. PARP-Inhibitor

Emerging evidence suggests that PTEN is involved in other functions beyond the PI3K/Akt pathway, namely DNA damage repair via homologous recombination [13,62,63].

Interestingly, in a phase II trial testing the PARP-inhibitor Talazoparib as monotherapy for BRCA(Breast Related Cancer Antigen) 1/2 wild-type HER2- BC patients, authors reported that tumors which exhibited response or stability harbored gene mutations in the homologous recombination pathway, including PTEN gene mutations [41].

## 4. Conclusions

Although there is some evidence for an association between PTEN functional status and both clinical outcome and response to various treatments, robust evidence is missing to properly establish its actual prognostic and/or predictive role in BC.

One of the major limitations is that PTEN assessment lacks consistency and reproducibility across studies in terms of type of assay (immunohistochemistry versus next-generation sequencing), antibodies for immunohistochemistry testing, scoring system (e.g., H-score, protein levels, % of positive cells), cutoffs for PTEN-loss definition, and origin of tumor samples (primary versus metastatic samples).

In addition, the clinical utility of PTEN-loss has been explored in cohorts of patients treated with considerably heterogeneous regimens, thus further increasing the complexity of the interpretation of available results.

To conclude, although currently unfulfilled, the potential of PTEN as a biomarker in BC is promising and deserves further investigation. Indeed, the optimal inter-observer and inter-antibody agreement of PTEN assessment, reported in the context of a prospective cohort of BC patients enrolled in a phase III randomized trial [29], appears reassuring and encourages further efforts in the context of well-designed and well-conducted biomarker-driven trials. Moreover, the recent finding that PTEN seems to be implicated in the control of tumor microenvironment and immune system leads to future potential perspectives for an immunotherapy-based therapeutic strategy.

## Figures and Tables

**Table 1:** Phase II and phase III clinical trials with translational analyses exploring the predictive role of chromosome ten (PTEN) in human epidermal growth factor receptor 2 (HER2)-negative breast cancer.

Drug Family	Agent	Trial	Phase	Population	Setting	Arms	N pts (Translational Analysis)	PTEN Analysis	Main Results
**mTOR Inhibitor**	Everolimus	TAMRAD [35]	II	HR+/HER2- MBC	AI-resistant	Tamoxifene + Everolimus Tamoxifene	30 25	IHC clone 138G6 Cell Signaling Technology (% and intensity: 1+, 2+, 3+)	No predictive role (TTP)
		BOLERO-2 [36]	III	HR+/HER2- MBC	AI-resistant	Exemestane + Everolimus Exemestane + Placebo	209 93	NGS (at least 1 mutation)	No predictive role (PFS)
								IHC (low-PTEN: H-score < 10)	No predictive role (PFS)
**AKT Inhibitor**	Ipatasertib	LOTUS [37]	II	TN MBC	First Line	Paclitaxel + Ipatasertib Paclitaxel + Placebo	54 47 (IHC) 49 (NGS)	IHC (low PTEN score 0 in at least 50% of tumor cells)	No predictive role (PFS)
								NGS (genetic-inactivating alterations)	mPFS of 9.0 m with ipatasertib versus 4.9 m with placebo (non-stratified HR 0.44, *p* = 0.041) in PIK3CA/AKT1/ PTEN-altered subgroup
		FARLAINE [38]	II	TN EBC	Neoadjuvant	CT + Ipatasertib CT + Placebo	Overall 132	IHC clone Ventana SP218 (low PTEN: score 0%–50% of tumor cells)	pCR rate 16% with ipatasertib versus 13% with placebo in PTEN-low population
								NGS (PTEN genomic alterations)	pCR rate 18% with ipatasertib versus 12% with placebo in PIK3CA/AKT1/PTEN-altered subgroup
	Capivasertib	PAKT [39]	II	TN MBC	First Line	Paclitaxel + Capivasertib Paclitaxel + Placebo	Overall 140	NGS (genetic alteration)	mPFS of 9.3 m with capivasertib versus 3.7 m with placebo in PIK3CA, AKT1 or PTEN-altered subgroup (HR 0.30, *p* = 0.01)
		FAKTION [40]	II	HR+/HER2- MBC	AI-resistant	Fulvestrant + Capivasertib Fulvestrant + Placebo	69 71	IHC	No predictive role (PFS)
**PARP Inhibitor**	Talazoparib	NCT02401347 [41]	II	HER2- MBC (plus other solid tumor)	Pretreated	Talazoparib	20 (13 BRCA1/2 wt MBC and 7 non- breast)	NGS (homologous recombination pathway: PTEN gene mutation)	3 pts had a RECIST response (ORR = 25%, 2 gPALB2, 1 gCHEK2/gFANCA/sPTEN) and 3 additional pts (gPALB2, sATR, sPTEN) had SD ≥ 6 m. (CBR = 50%)
**Endocrine Therapy**	Anastrozole/Fulvestrant	CARMINA 02 [42]	II	HER2- EBC	Neoadjuvant	Anastrozole Fulvestrant	Overall 55 (RNA) 89 (NGS)	gene expression (PTEN-related encoding gene TPTE)	PTEN-related encoding gene TPTE significantly overexpressed in responders but not in non-responders

**Abbreviations**: HR+, hormone-receptor positive; MBC, metastatic breast cancer; AI, aromatase inhibitor; IHC, immunohistochemistry; TTP, time-to-progression; NGS, next-generation sequencing; (m)PFS, (median)progression-free survival; TN, triple-negative; AKT, protein kinase B; EBC, early breast cancer; CT, chemotherapy; pCR, pathologic complete response; HR, hazard ratio; ORR, overall response rate; SD, stable disease; m., months; PARP, poly ADP-ribose polymeraseCHECK2, checkpoint kinase 2; FANCA, Fanconi Anemia group A protein; CBR, clinical benefit rate.

**Table 2:** Phase II and phase III clinical trials with translational analyses exploring the predictive role of PTEN in HER2-positive breast cancer.

Treatment Strategy	Agent	Trial	Phase	Population	Setting	Arms	N pts (Translational Analysis)	PTEN Analysis	Main Results
**Single anti-HER2 blockade**	Trastuzumab	NCT00133796 [43]	II	EBC	Neoadjuvant	CT + Trastuzumab	35	IHC (protein level: 0 versus 1+ versus 2+ versus 3+)	pCR rates in low-PTEN versus high-PTEN: 15.4% versus 44.4%
		GeparQuattro [44]	III	EBC	Neoadjuvant	CT (anthra-taxane) + Trastuzumab 2 arms with CT (anthra-taxane-cape) + Trastuzumab	Overall 108	automated quantitative immunofluorescence (PTEN-low <60.1)	pCR rates in high-PTEN versus low-low PTEN: 57.1% versus 27.6%
		N9831 [28]	III	EBC	Adjuvant	CT alone CT + sequential trastuzumab CT + concurrent trastuzumab	601 650 551	IHC (PTEN+: >0% invasive cells with ≥1+ cytoplasmatic staining; examination of alternate cut-points)	No predictive role with different cut-points (DFS)
		BCIRG-006 [33]	III	EBC	Adjuvant	CT (anthra) + Trastuzumab CT (non-anthra) + Trastuzumab CT	402 405 394	IHC clone 9559 Cell Signaling Technology (protein level: 0 versus 1+ versus 2+ versus 3+)	No predictive role (DFS, OS)
	Lapatinib	EGF103009 [45]	II	MBC	Refractory or recurrent after anthracycline-containing regimen in the adjuvant or metastatic setting	Lapatinib	30	IHCclone by Cascade Bioscience (protein level: 0 versus 1+ versus 2+ versus 3+)	No predictive role (ORR)
		EGF104535 [31]	III	MBC	First-line	CT + Lapatinib CT + placebo	180 175	IHC clone 138G6 Cell Signaling Technology (protein level: 0 versus 1+ versus 2+ versus 3+; two alternative cutoffs for PTEN-low: 0/1+ or 0)	No predictive role (PFS, OS)
**Dual anti-HER2 blockade**	Trastuzumab + Pertuzumab	Cleopatra [30]	III	MBC	First-line	CT + Trastuzumab + Pertuzumab CT + Trastuzumab + Placebo	Overall 497	IHC clone AF847; R and D Systems (modified H-scores: 0–400, for membrane, cytoplasm and nucleus; low versus high: median value adopted as cutoff)	No predictive role (PFS, OS)
		TRYPHAENA [46]	II	EBC	Neoadjuvant	3 arms of Pertuzumab + Trastuzumab + CT (different CT regimens)	Overall 225	IHC clone by Cell Signalling (modified H-score: 0–400, nuclear compartment; low versus high: median value adopted as cutoff)	No predictive role (pCR)
		NeoSphere [47]	II	EBC	Neoadjuvant	CT + Trastuzumab CT + Pertuzumab + Trastuzumab CT + Pertuzumab Trastuzumab + Pertuzumab	95 95 85 98	IHC (modified H-score: 0–400, cytoplasmatic compartment; low versus high: median value adopted as cutoff)	No predictive role (pCR)
		Aphinity [34]	III	EBC	Adjuvant	CT + Trastuzumab + Pertuzumab CT + Trastuzumab + Placebo	Overall 939	DNA analysis for PI3K pathway alterations	Trend towards decreased benefit from pertuzumab in patients with PTEN/AKT/PIK3CA alterations (NS)
	Trastuzumab + Lapatinib	NCT00206427 [43]	II	EBC	Neoadjuvant	CT + Trastuzumab + Lapatinib	49	IHC clone AF847, R and D Systems (protein level: 0 versus 1+ versus 2+ versus 3+)	pCR rates in low PTEN versus normal-PTEN: 92.3% versus 41.2%
		NCT00524303 [48]	II	EBC	Neoadjuvant	CT + Trastuzumab CT + Lapatinib CT + Trastuzumab + Lapatinib	Overall 49	NA	No predictive role (pCR)
		Neo-ALTTO [29]	III	EBC	Neoadjuvant	Trastuzumab Lapatinib Trastuzumab + Lapatinib	Overall 429	IHC clone 6H2.1 DAKO and clone 138G6 Cell Signaling Technology (H score system: PTEN-low H-score < 50 in invasive tumor cell compartment; analysis of alternative cut-point H-score ≤ 10; 2 observers)	No predictive role (pCR)
		CHER-LOB [49]	II	EBC	Neoadjuvant	CT+ Trastuzumab CT + Lapatinib CT + Trastuzumab + Lapatinib	Overall 121	IHC clone 28H6 Novocastra (PTEN loss: staining < 10% cancer cells)	No predictive role (pCR)
		ICORG 10-05 [50]	II	EBC	Neoadjuvant	CT + Trastuzumab CT + Lapatinib CT + Trastuzumab + Lapatinib	21 6 18	IHC clone 6H2.1 Dako (protein level: 0 versus 1+ versus 2+ versus 3+)	No predictive role (pCR)
		TBCRC006 [51]	II	EBC	Neoadjuvant	Trastuzumab + Lapatinib	59	IHC clone D4.3 Cell Signaling (H-score. PTEN low versus high: H-score <100 versus ≥100)	pCR rates in high-PTEN versus low-PTEN: 32% versus 9%
**Drug conjugate**	TDM1	Emilia [52]	III	MBC	Previous treatment with Trastuzumab and a taxane	TDM1 CT + Lapatinib	134 137	IHC clone 138G6 Cell Signaling Technology (cytoplasmic PTEN expression: none versus decreased versus slightly decreased versus equivalent versus increased)	Absent or decreased tumor PTEN expression associated PFS benefit with T-DM1 relative to that with CT + Lapatinib
		TH3RESA [32]	III	MBC	≥2 previous anti-HER2 regimens (including Trastuzumab and Lapatinib in the advanced setting)	TDM1 TPC	247 111	IHC clone 138G6 Cell Signaling Technology (H-score: 0–400; low versus high: median adopted as cutoff = 200)	No predictive role (PFS)
		Marianne [53]	III	MBC	First-line	CT + Trastuzumab TDM1 TDM1 + Pertuzumab	182 181 179	IHC clone 138G6 Cell Signaling Technology (protein level: 0 versus 1+ versus 2+ versus 3+; H-score: median value adopted as cutoff, cytoplasmatic compartment)	No predictive role (PFS)

**Abbreviations:** EBC, early breast cancer; CT, chemotherapy; IHC, immunohistochemistry; pCR, pathologic complete response; DFS, disease-free survival; anthra, anthracycline-containing regimen; non-anthra, non-anthracycline containing regimen; OS; overall survival; cape, Capecitabine; MBC, metastatic breast cancer; PFS, progression-free survival, ORR, overall response rate; NA, not applicable; NS, not-significant; TDM1, trastuzumab-emtansine; TPC, treatment of physician’s choice.
